# Detection of inborn error of metabolisms by urine organic acid GC-MS in Southern China

**DOI:** 10.1186/1687-9856-2013-S1-P180

**Published:** 2013-10-03

**Authors:** MinYan Jiang, Li Liu, HuiFen Mei, XiuZhen Li, Jing Cheng, YanNa Cai, Wen Zhang, XiaoJian Mao, ZhiKun Lu

**Affiliations:** 1 Department of Endocrinology and Metabolisms, Guangzhou Women and Children’s Medical Center, Guangzhou, Guangdong, China

## Background

Inborn error of metabolisms (IEM) have been detected worldwide using gas chromatography mass spectrometry since 1980s, but few related date presently exit in Southern China. This study aimed to evaluate the prevalence, spectrum and clinical presentation of IEM in Southern China.

## Method

From January 2009 to March 2012, 16075 urine samples were collected from patients with developmental delay, seizures, vomiting and metabolic acidosis in Guangzhou Women and Children’s Medical Center.

## Results

We diagnosed 148 cases of IEM by urine GC-MS analysis, including 97 cases of organic acid disorders, 41 cases of amion acid disorders and 10 cases of fatty acid oxidative disorders. Methylmalonic aciduria (MMA) was most common(48 cases), followed by urea cycle disorder (21 cases), phenylketonuria (20 cases),propionic aciduria(11 cases), multiple carboxylase deficiency(8 cases), glutaric aciduria type I (7 cases), oxoproliemia(7 cases), isovaleric aciduria(6 cases),glutaric aciduris type II and Short chain acyl-CoA dehydrogenase deficiency(4 cases),3-hydroxy-3-methylglutaric aciduria(3 cases), amionadipic aciduria(2 cases),maple syrup urine disease(2 cases), very long-china acyl-CoA dehydrogenase deficiency(2 cases), Malonic aciduria(1 case), Canavan disease(1 case) and mevalonic aciduria(1 case). Average age at diagnosis was 18 months. Prompt therapy was taken, including dietary and medicine treatment. Clinical improvements were observed in more than half of the patients.

## Conclusion

In Southern China, the majority of IEM were organic acid disorders and amino acid disorders. Fatty acid oxidation disorders were relatively rare. The age at diagnosis was early and incidence of IEM gradually decreased with the age. Urine GC-MS was an important technique to diagnose IEM, which helped to improve patients’ prognoses.

